# Using Virtual Reality Pablo Gaming in the Post-Operative Rehabilitation of Breast Cancer Patients: Randomized Controlled Trial

**DOI:** 10.3390/jcm13247609

**Published:** 2024-12-13

**Authors:** Ahmed Abdelmoniem Ibrahim, Sobhy M. Aly, Ahmed S. A. Youssef, Mohamed Marzouk Mohamed Ragab, Hisham M. Hussein

**Affiliations:** 1 Department of Physical Therapy, College of Applied Medical Sciences, University of Ha’il, Ha’il 81451, Saudi Arabia; hm.hussein@uoh.edu.sa; 2 Department of Biomechanics, Faculty of Physical Therapy, Cairo University, Giza 12613, Egypt; smali@nu.edu.sa; 3 Department of Medical Rehabilitation Sciences, College of Applied Medical Sciences, Najran University, Najran 66462, Saudi Arabia; 4 Department of Basic Sciences, Faculty of Physical Therapy, Beni-Suef University, Beni-Suef 62511, Egypt; dr.ahmedsameer@pt.bsu.edu.eg; 5 Department of Basic Sciences for Physical Therapy, Faculty of Physical Therapy, Cairo University, Giza 12613, Egypt; mohamed.ragab@cu.edu.eg

**Keywords:** tumor, surgery, Pablo, physical training

## Abstract

**Background/Objectives:** Surgical treatment of breast cancer may lead to physical and psychological side effects. Exercises, especially those aided by virtual reality (VR), can improve both physical and psychological dysfunctions. To explore the effects of exercises using VR through Pablo games Technology on the function, grip strength, wrist ROM, fatigue, pain, activities of daily living (ADLs), and anxiety among post-operative breast cancer females. **Methods:** Forty post-operative breast cancer females participated in the current study: nineteen in the control group (CG), who received a standard treatment consisting of upper limb exercises plus intermittent compression therapy, and twenty-one participants assigned to the Pablo group (PG), who received the standard treatment plus additional training using the Pablo game training system. The intervention period was eight weeks long. The outcome measures were function, grip strength, wrist ROM, fatigue, pain, activities of daily living, and anxiety. Data were obtained at the baseline, after eight weeks, and at two months follow-up. **Results:** There were statistically significant declines in pain and fatigue, while there were statistically significant improvements in ADLs, grip strength, function, and ROM post-intervention and at two months follow-up in both groups (*p* < 0.001). Between-group comparisons demonstrated a statistically significant decrease in pain, anxiety, and fatigue and statistically significant improvements in function, ADLs, grip strength, and ROM in favor of the PG post-treatment and at the follow-up (*p* < 0.001). **Conclusions:** Adding VR using the Pablo game training system to the standard rehabilitation of post-surgical breast cancer patients can further improve their function, hand grip, wrist ROM, fatigue, pain, and ADLs.

## 1. Introduction

The World Health Organization (WHO) states that breast cancer (BC) accounts for 16% of all cancers in women; every year, more than one million women worldwide are diagnosed with breast cancer [1]. BC is considered the most common disease among females. With the recent advances in diagnosis and treatment technology, a growing number of females receive early diagnosis and treatment. Hence, survival rates are rising dramatically [2].

The fundamental treatment options for BC include surgery, chemotherapy, radiotherapy, and hormone therapy. Despite being effective, these treatments have several side effects such as limited range of motion (ROM), muscle weakness, fatigue, lymph edema, decreased functional activity, and depression, which have been reported in a previous study [3]. These adverse consequences significantly impede patients’ ability to adhere to treatment regimens, perform daily tasks independently, and maintain a high quality of life [4].

Several evidence-based studies have shown the benefits of physical rehabilitation in addressing the side effects of BC. These benefits include improved physical function, muscle power, and psychological well-being [5,6,7,8]. To further augment the benefits of physical rehabilitation, virtual reality (VR) has been increasingly incorporated into the field of rehabilitation [4]. VR can be described as a form of artificial intelligence (AI) originally introduced in the 1960s and gradually developed throughout the 20th century [9]. Recently, significant technological developments have made the use of AI applications in the medical field accessible [10,11].

In physical rehabilitation with VR, users can experience a more immersive experience using multiple senses (vision, hearing, and touch) by combining computerized systems with multi-dimensional graphics [12], and it is highly motivating for patients to engage in therapeutic intervention [13,14].

Recent studies have reported promising results from the introduction of VR (Virtual Therapeutic Garden) to BC rehabilitating. For example, a recent pilot study reported reduced depressive symptoms and improved psychological wellness; however, no between-group differences were reported regarding the physical activity level [15]. Another study conducted by Chirico and colleagues assessed the efficacy of VR using head-mounted glasses in relieving psychological symptoms in BC females post-chemotherapy. Symptoms such as fatigue and anxiety demonstrated greater improvement in patients who received the VR rehabilitation protocol [16]. In a trial conducted by Feyzioğlu and colleagues, improvements in pain, muscle strength, ROM, and function were evident in BC patients who used VR during the early post-operative phase [17]. 

Pablo is a type of VR application that functions as a game-based rehabilitation system, consisting of a handheld device, a multi-ball system, and a multi-board system. It trains the visuomotor system to help the patient to learn motor skills. Additionally, it can be used to measure wrist ROM and grip strength [18,19]. Concurrently, it is used for visuomotor training. When using the Pablo system, users are provided with both visual and audio feedback regarding their visuomotor training. This multimode feedback reflects task completion and progress using a rewarding mechanism. Game-based training has been demonstrated to increase motivation and attention in addition to functional and quantitative scores, which ultimately help to improve patient performance [20].

Few trials related to the use of VR in the treatment of patients with BC are available, and Pablo was not used in any of them. These studies presented several limitations, such as sample size, including Czech et al.’s study [15], where only 16 patients were included. There was also a lack of a control group on many occasions [1,15,21,22]. Finally, a short intervention duration is evident in many studies, ranging from a single session [23] to two weeks [15], while other researchers applied interventions during chemotherapy sessions [1,16]. Compared to previous studies, our study had several unique features, including a relatively acceptable sample size that was pre-calculated, a relatively long study duration, the use of the Pablo system for training and assessment, and the implementation of a follow-up design, and these differences could explain the variation in reported findings between our work and previous research.

As a result of a recent review, it is evident that there are no well-designed trials available. This results in low-quality evidence and an inability to reach a conclusive decision about the effectiveness of VR in the management of BC patients [14,24]. This study sought to address the impact of adding VR-augmented exercises—through Pablo’s game—to the standard rehabilitation on function, grip strength, ROM, fatigue, pain, activity levels, and anxiety in post-operative BC patients.

This study hypothesizes that adding Pablo’s game exercises to the standard rehabilitation of the post-operative BC patients might improve function, grip strength, ROM, fatigue, pain, activity levels, and anxiety.

## 2. Methods

This double-blinded randomized control trial design was conducted between September 2023 and March 2024 in a specialized rehabilitation center for cancer patients located in Al Qassim, Saudi Arabia. This study followed the guidelines of the Declaration of Helsinki and was reported according to the CONSORT statement for reporting randomized controlled trials. Ethical clearance was granted by the institutional review board of the University of Ha’il, Saudi Arabia (NO: H-2023-366). Informed consent was provided by each participant before starting the trial.

### 2.1. Participants

The inclusion criteria were (a) female patients aged 20 to 60 years, (b) had either stage I or II BC, (c) underwent surgical treatment through radical mastectomy, that is the removal of the whole breast, all lymph nodes located under the arm, and the chest wall muscles under the breast [25] or modified radical mastectomy by removing the breast, including the nipple-areola complex, the axillary lymphatic system, and the skin covering the tumor area [26], (d) able to use the Pablo device, (e) patients underwent preoperative education and advice regarding post-operative rehabilitation program, and (f) the patient was ambulatory and fully active (had a score of ≤2 on the Eastern Cooperative Oncology Group) [27].

The exclusion criteria were (a) medical conditions that restrict her sharing in the training program, such as anemia, unstable cardiovascular diseases, uncontrolled diabetes, neuromuscular disorders, (c) extensive brain or bone metastases, and (d) any contraindications given by the physician.

### 2.2. Sample Size

The sample size was calculated in advance using G*POWER software (3.1.9.7; Heinrich-Heine-Universität Dusseldorf, Dusseldorf, Germany). The DASH score was used for the calculation, where the mean and SD were retrieved from a previous study [17]. The calculation results with power = 0.80, α = 0.05, and effect size = 0.30 suggested a sample size of 50 (25 in each group) for this study. To compensate for a possible 20% dropout, 30 patients in each group were recruited.

### 2.3. Interventions

All participants received exercise therapy plus intermittent compression. The Pablo group (PG) received additional exercises using the Pablo training system. The intervention program was conducted three times per week for eight weeks.

#### 2.3.1. Exercises

The standard exercise program consisted of five exercise tasks. Each exercise was performed with 2 sets × 15 repetitions per session. The total exercise duration was approximately 15 min. Exercises were stopped if the desired repetitions were reached or signs of fatigue were evident (e.g., decreased performance, weakness, or jerky movements). The exercises used in this study included pumping exercises for the upper limbs, pendulum exercises (anterior–posterior and medial–lateral), shoulder ROM exercises, arm raises with clasped hands, and wrist ROM exercises in all directions (flexion and extension exercises, as well as ulnar and radial deviation).

#### 2.3.2. Intermittent Compression

An electronic intermittent compression therapy unit (Care Pump Expert 8, BardoMed Sp. z o.o. ul., Kraków, Poland) was used to apply pneumatic compression to the affected upper limb. The parameters of the intermittent compression were a pressure of 60 mmHg applied from distal to proximal in a sequential manner for 15 min [28].

#### 2.3.3. Pablo© Handle Training

One-dimensional games using handheld devices were performed. Five different games (Recycle, Firefighters, Shooting Cans, Balloon, and Apple Hunter) were played in each session. The duration for each game was 3 min, resulting in a total training time of 15 min [18].

Each game was randomly played during the sessions, and the difficulties depended on the repetition, speed, and time of the games, as follows: (a) Recycle: using a gripper to pick up various waste items and place them in the appropriate container—reaching and preserving the necessary degree of motion or strength; (b) Firefighters: extinguishing flames using a water jet that is controlled precisely—achieving and maintaining a certain level of strength and/or motion; (c) Shooting Cans: pulling the trigger at the right time to shoot cans moving across the screen—timely activation of strength and/or motion impulses; (d) Balloon: moving a balloon through tracks and overcoming obstacles with dynamic motion; (e) Apple Hunter: catching apples in the basket on the screen [18].

### 2.4. Outcome Measures

This study examined three main outcome measures (function, handgrip strength, and wrist ROM) and four secondary outcomes (fatigue, pain intensity, activities of daily living (ADLs), and anxiety). These measures were obtained at baseline, post-intervention, and at two months follow-up. Assessments were conducted by an experienced physical therapist blinded to the group allocation.

#### 2.4.1. Upper Limb Functioning

The assessment of upper extremity functioning was assessed using a validated Arabic version of the function of the Arm, Shoulder, and Hand (DASH) questionnaire [29]. This scale consists of 30 items, with each assigned a number between 1 (no difficulty) and 5 (inability). Higher cumulative scores indicate a higher level of functional impairment. The cumulative score ranges from 0 to 100 points [30].

Hand grip and wrist ROM.

Pablo (Tyromotion GmbH, Graz, Austria) was used to assess the handgrip strength and wrist joint ROM. The patients assumed a sitting position with their elbows flexed 90 degrees and their forearms neutrally positioned [18].

#### 2.4.2. Fatigue

Fatigue was measured using the Multidimensional Fatigue Inventory (MFI), which is a self-reporting tool consisting of 20 items designed to measure fatigue for cancer patients. The patients were required to respond to each item based on their perceived fatigue levels. Responses were typically scored ranging from 1 to 5, with 1 indicating “never” experiencing the stated fatigue-related symptom and 5 indicating “very much” or “always” experiencing it. Higher scores represent greater fatigue. The MFI is a valid and reliable instrument for assessing fatigue across different populations and contexts [31,32].

#### 2.4.3. Pain Intensity

Pain was assessed using the Visual Analog Scale (VAS), which is simple, widely recognized, and reliable [33]. The VAS includes a 10-point scale (0 being no pain and 10 being the greatest pain). This scale provides clinicians with valuable insights into the subjective experience of pain, allowing for accurate assessment and monitoring of pain intensity over time [34].

#### 2.4.4. Activities of Daily Living (ADLs)

The Barthel Index (BI) was used to assess ADLs, The scale was designed to measure an individual’s ability to perform basic daily tasks and it is valid and reliable [35]. Scores vary from 0 to 100, where higher scores represent greater performance. There are several domains measured by the BI: feeding, grooming, bathing, dressing, bowel control, bladder control, toileting, transfers from bed to a chair, ambulation, and stair climbing [36].

#### 2.4.5. Anxiety

Anxiety level was assessed using the State Anxiety Inventory (SAI), a well-established and widely adopted tool for evaluating anxiety [37]. The SAI comprises 20 items, with a 4-point scale for each item (1 = almost never, 2 = occasionally, 3 = most of the time, and 4 = almost always). Participants provide responses based on the extent to which they experience feelings of anxiety. The SAI score ranges from 20 to 80, demonstrating higher anxiety [38]. This comprehensive instrument provides valuable insights into the current state of anxiety experienced by individuals, allowing healthcare professionals to tailor interventions and support strategies accordingly [23].

### 2.5. Allocation, Concealment, and Blinding

Following the initial screening, the included patients were randomly allocated into two groups the control group (CG) and the Pablo group (PG). The allocation procedure was conducted by the senior author who was neither involved in the assessment nor the treatment. The allocation sequence was performed using Statistical Package for Social Sciences (SPSS) a random number generator. The assessor, the patients, and the statistician were blind to the allocation.

### 2.6. Statistical Analysis

Statistical analysis was conducted by using SPSS version 25 for Windows. An unpaired *t*-test and chi squared were conducted to compare group characteristics. The data normality was tested using the Shapiro–Wilk test, while Levene’s test was used to assess the equality of variances. A mixed MANOVA was used to test the effect of treatment on the measured outcomes. Multiple post hoc tests were conducted using Bonferroni correction. The level of significance was set at *p* < 0.05.

## 3. Results

**Figure 1:**
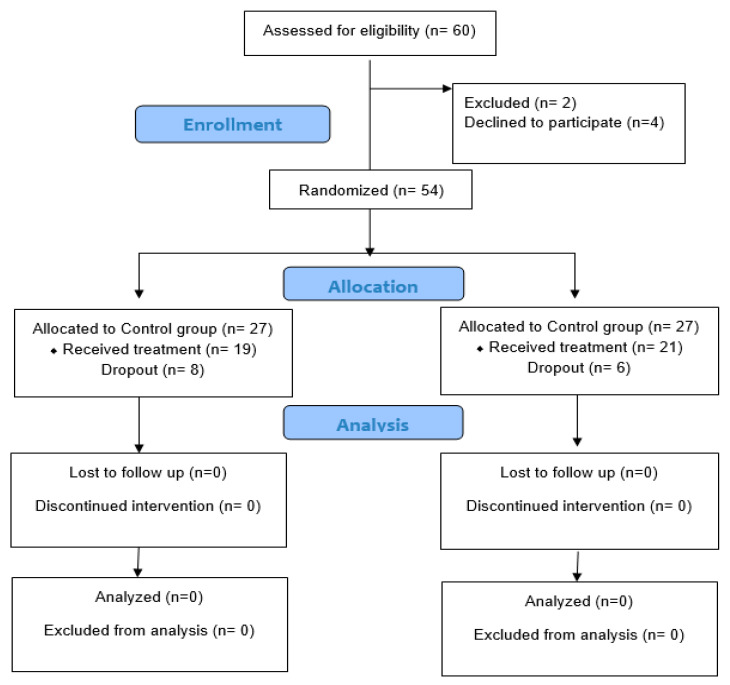
Study flow chart.

After the screening, sixty females with post-operative breast cancer were allocated for the study, six women were excluded, and fifty-four women joined the trial (twenty-seven women for each group). Eight females from the CG and six from the PG were not allocated intervention due to personal and social issues (Figure 1). Forty women (twenty-one PG, and nineteen CG) completed the study with 100% commitment to the treatment sessions. No intervention-related adverse effects were reported in both groups and no patient was lost during follow-up.

The basic characteristics of the participants are illustrated in Table 1. All demographic data were comparable between groups (*p* > 0.05). The participants were subjected to either radical or modified radical mastectomy operations. Radiotherapy was performed for 33% of patients, chemotherapy for 30% of patients, and radiation with chemotherapy for 37% of patients.

There was a statistically significant interaction of treatment * time (F = 25.58, *p* = 0.001, ηp2 = 0.94). There were also statistically significant main effects for time (F = 211.99, *p* = 0.001, ηp2 = 0.99) and treatment (F = 24.35, *p* = 0.001, ηp2 = 0.86).

### 3.1. Between-Group Comparison

A statistically significant difference was observed in pain, function, anxiety, fatigue, ADLs, and grip strength between both groups post-treatment and at follow-up (*p* < 0.001). At two months follow-up, significant difference between both groups in wrist ROM (*p* < 0.05) (Table 2).

### 3.2. Within-Group Comparison

Statistics showed that there were significant differences in pain, function, fatigue, ADLs, and grip strength post-intervention and at two months follow-up compared to baseline values (*p* < 0.001) and at follow-up compared to post-treatment (*p* < 0.01).

In the PG, there was a statistically significant decrease in participants’ anxiety level post-treatment and at follow-up compared to baseline and at follow-up compared to post-treatment (*p* < 0.001). Regarding CG, there were no statistical differences in anxiety level post-treatment (*p* = 0.25), while statistically significant differences were observed at two months follow-up compared to baseline and post-treatment (*p* < 0.001).

The wrist ROM demonstrated statistically significant differences post-treatment and at two months follow-up in both groups (*p* < 0.001), while in the PG, statistically significant differences were evident upon comparing the follow-up and post-treatment values (*p* < 0.001) (Table 3 and Table 4).

## 4. Discussion

The current trial was conducted to explore the additional benefits that might be gained when Pablo gaming is added to the standard physical therapy for the patient’s post-surgical treatment for BC. We found that using VR game-based rehabilitation produces more significant positive effects when compared to standard physical therapy care concerning function, grip strength, wrist ROM, fatigue, pain, activity level, and anxiety.

In the current study, the Pablo (Tyro-motion) device was used to conduct VR training games. The training was performed through interactive games while assessing different outcomes such as handgrip strength, and ROM for all upper limb joints [39,40]. To the best of the authors’ knowledge, this study might be considered the first randomized controlled trial that used Pablo games in the rehabilitation of post-operative BC patients. So, the comparisons of the current work with previous literature might be hard. However, different types of VR devices were used in previous work [16,41].

Feyzioğlu et al. applied Xbox Kinect-based VR on 40 post-operative breast cancer female patients for 6 weeks of training sessions, results were significant regarding (pain, ROM, and grip strength) when compared to standard physiotherapy [41]. These findings support the results of the current study.

Another form of VR gaming technology using head-mounted glasses (VuzixWrap 1200VR) with a head motion tracking system was applied by Chirico and colleagues on 64 BC female patients under chemotherapy. The control group in this study received music therapy, results revealed that anxiety, depression, and fatigue seem to be better relieved with VR than with music therapy [16]. Similarly, Atef and colleagues have used Nintendo Wii^®^ video games on 30 post-mastectomy female patients (15 for VR and 15 for PNF), and the program, which lasted four weeks, proved that both Nintendo Wii^®^ video games and proprioceptive neuromuscular facilitation were effective interventions for edema after unilateral mastectomy. A reduction in edema could positively impact the ROM and functional ability of the patient. However, the ability to provide feedback added to the superiority of the Nintendo Wii^®^ video games in terms of motivation [42]. Unfortunately, the current study did not include limb edema as an outcome measure.

Many previous studies investigated functioning in BC patients after using VR [15,21,22,41,42]. In contrast to the current study findings, the use of VR technology in previous studies seems to have no superior effects compared to comparator intervention on function. The VR devices that were used did not show any superior effects in terms of function [15,41]. House and colleague’s study did not conduct between-group comparisons, only within-group comparisons in which the VR and standard treatment groups demonstrated significant changes in upper limb functioning, as indicated by the UEFI-20 scale [21]. Similarly, Atif et al. [42] reported a lack of significant differences between the VR and proprioceptive neuromuscular facilitation (PNF) techniques in improving functioning.

Regarding hand grip strength and wrist ROM, it was measured by Pablo (Tyro motion) results showed significant positive effects when compared to standard physical therapy. Contradicting findings were reported regarding hand grip strength and wrist ROM. Feyzioğlu’s study assessed handgrip strength using a handheld dynamometer and assessed ROM using an electrical goniometer after applying Xbox Kinect-based VR to the experimental group and standard physiotherapy for the control group. Both groups demonstrated similar improvements in terms of hand grip strength and ROM [41]. On the other hand, Chan and colleagues reported significant improvements in shoulder abduction and flexion ROM after early rehabilitation for 6 weeks [22].

Fatigue was one of the outcome measures in Chirico et al.’s study [16]. A comparison with music therapy and standard treatment revealed that VR was better as a distraction therapy, significantly improving resistance to fatigue in BC patients receiving chemotherapy. This finding aligns with our results; however, unlike in Chirico et al.’s study, our intervention was not performed at the time of receiving chemotherapy.

In the current study, pain was measured and results showed significant positive effects in the VR group (Pablo) compared to standard physical therapy. Pain intensity was assessed in previous studies using VR for BC rehabilitation. House et al. found a decline in pain intensity when using Bright Arm Duo therapy as VR training; however, one of six patients demonstrated a statistically significant reduction in pain measures [21]. In another study conducted on 80 breast cancer patients, a significant improvement in pain scores was evident when VR training (immersive VR) was added to morphine medication [23]. In contrast, Feyzioğlu et al. [41] did not report any additional benefits regarding pain scores after the application of Kinect-based VR in comparison to standard treatment.

Regarding ADL, House et al. assessed ADLs in a single group of post-surgical BC patients. They reported the ability of patients to use their upper extremities in various ADLs using the UEFI-20. Unfortunately, no control group was implemented [21]. However, there was a significant improvement after the application of VR when compared to the experimental group, which supports our findings, but we used the Barthel Index for measuring ADL.

Anxiety levels showed significant improvement after using VR compared to music or standard care of BC patients receiving chemotherapy [16]. Another study found that immersion VR plus morphine was superior to morphine alone in alleviating anxiety after a single session that was applied during a chemotherapy session [23]. Buche et al. conducted a study to test the impact of different types of virtual reality on post-operative BC patients, and results showed better anxiety scores measured by the State Anxiety Inventory (SAI) Scale when compared to music therapy [1]. This supports the current study findings in improving anxiety levels in post-operative BC patients after using VR training (Pablo). Chirico et al. conducted a review of 19 trials and proved that VR therapies helped cancer patients feel better emotionally and experience less suffering connected to their disease, whether it was from hospitalization, unpleasant procedures, or chemotherapy [43].

The clinical implication of our trial is that the Pablo system was useful in the rehabilitation of BC patients and in measuring wrist range of motion and hand grip strength. Additionally, our study offers a novel perspective on Pablo-based game therapy, which incorporates accurate evaluation and engaging training techniques to address pain, functioning, fatigue, anxiety, and daily activity levels in breast cancer patients.

We have some limitations in our study, which could be explored in a future study. The primary limitation was a lack of long-term follow-up, dropout of participants during randomization, and patient blinding. Second, we did not include different types of surgical procedures in the inclusion criteria, only radical mastectomy. Also, we did not assess the effects of different types of virtual reality to identify the effective treatment types, frequency, and duration for post-operative breast cancer patients. Additionally, as we combined Pablo’s training and physical therapy, we were unable to differentiate the effects of each individually on outcome measures.

Future research is required to provide a thorough qualitative analysis of patients’ experiences during Pablo’s game rehabilitation sessions with long-term follow-up. Research should be applied to measure the effectiveness of Pablo’s game rehabilitation along with other types of breast cancer surgery and to compare it with other virtual reality. Additionally, we shall examine the effect of Pablo’s game on different types of breast cancer surgery.

## 5. Conclusions

Adding Pablo games to the rehabilitation program of post-operative BC patients, who suffered stage I or II cancer, improved their pain, function, strength, anxiety, fatigue, and daily activity levels. The application of Pablo’s gaming technology for therapy led to the creation of an entirely new organizational model.

## Figures and Tables

**Table 1:** Characteristics of the participants.

	PG	CG	MD	t-Value		*p*-Value
	Mean ± SD	Mean ± SD				
**Age (years)**	47 ± 3.94	48 ± 4.60	−1	−0.56		0.57
**BMI (kg/m^2^)**	28.05 ± 3.04	28.80 ± 2.84	−0.75	−0.81		0.42
**Type of surgery, n (%)**						
**Radical mastectomy**	8 (38%)	5 (26%)	χ^2^ = 0.63		0.43	
**Modified radical mastectomy**	13 (62%)	14 (74%)				
**Tumor stage, n (%)**						
**Stage I**	11 (52%)	12 (63%)	χ^2^ = 0.47		0.49	
**Stage II**	10 (48%)	7 (37%)				
**lymph node involvement, n (%)**						
**N0**	9 (43%)	11 (58%)	χ^2^ = 0.94		0.63	
**N1**	8 (38%)	5 (26%)				
**N2**	4 (19%)	3 (16%)				
**Adjuvant therapy, n (%)**						
**Radiation**	7 (33%)	6 (32%)	χ^2^ = 2.98		0.22	
**Chemotherapy**	4 (19%)	8 (42%)				
**Radiation + chemotherapy**	10 (48%)	5 (26%)				

**PG**, Pablo group; **CG**, control group; **SD**, standard deviation; **MD**, mean difference; **χ^2^**, chi squared value; ***p*-value**, probability value; **n**, number; **%**, percentage; **BMI**, body mass index.

**Table 2:** Comparison of pain, function, anexity, fatigue, ADLs, ROM, and grip strength values between groups.

*Outcomes*		PG	CG	MD	95% CI		*p*-Value *	ηp2
		Mean ± SD	Mean ± SD		Lower Bound	Upper Bound		
**Pain** **(VAS)**	**Pre-treatment**	6.95 ± 1.07	6.79 ± 1.23	0.16	−0.57	0.90	0.65	0.005
	**Post-treatment**	4.33 ± 0.91	5.26 ± 1.20	−0.93	−1.61	−0.25	0.008	0.17
	**Follow-up**	2.71 ± 1.01	4.42 ± 0.96	−1.71	−2.34	−1.07	0.001	0.44
**Function** **(DASH)**	**Pre-treatment**	59.29 ± 5.62	57.42 ± 3.58	1.87	−1.19	4.92	0.22	0.03
	**Post-treatment**	38.10 ± 4.16	49.16 ± 3.72	−11.06	−13.60	−8.53	0.001	0.67
	**Follow-up**	29.05 ± 4.49	46.26 ± 3.43	−17.21	−19.79	−14.64	0.001	0.82
**Anexity (SAI)**	**Pre-treatment**	37.05 ± 4.62	35.26 ± 5.36	1.79	−1.41	4.98	0.26	0.03
	**Post-treatment**	30.48 ± 4.49	33.84 ± 5.07	−3.36	−6.43	−0.31	0.03	0.12
	**Follow-up**	25.81 ± 3.54	30.89 ± 4.70	−5.08	−7.73	−2.44	0.001	0.28
**Fatigue (MFI)**	**Pre-treatment**	51.81 ± 7.09	52.26 ± 7.50	−0.45	−5.13	4.22	0.84	0.001
	**Post-treatment**	40.24 ± 4.83	46.42 ± 7.14	−6.18	−10.05	−2.32	0.003	0.22
	**Follow-up**	29.48 ± 4.80	41.53 ± 6.65	−12.05	−15.74	−8.36	0.001	0.54
**Barthel Index for** **(ADLs)**	**Pre-treatment**	61.43 ± 5.73	62.89 ± 8.05	−1.46	−5.91	2.97	0.51	0.01
	**Post-treatment**	79.76 ± 6.22	72.63 ± 7.52	7.13	2.73	11.53	0.002	0.22
	**F** **ollow-up**	86.19 ± 4.98	75 ± 6.87	11.19	7.38	15.00	0.001	0.48
**Flexion ROM**	**Pre-treatment**	77.67 ± 2.56	78.79 ± 2.25	−1.12	−2.67	0.43	0.15	0.05
	**Post-treatment**	84.43 ± 2.18	84.47 ± 2.29	−0.04	−1.48	1.39	0.95	0.001
	**Follow-up**	87.43 ± 1.75	83.74 ± 2.26	3.69	2.41	4.98	0.001	0.47
**Extension ROM**	**Pre-treatment**	72.19 ± 2.96	73.16 ± 3.15	−0.97	−2.92	0.99	0.32	0.02
	**Post-treatment**	79.14 ± 3.99	78.42 ± 2.79	0.72	−1.51	2.95	0.51	0.01
	**Follow-up**	81.05 ± 3.11	78.79 ± 2.87	2.26	0.33	4.18	0.02	0.13
**Grip strength**	**Pre-treatment**	13.90 ± 1.73	13.21 ± 1.13	0.69	−0.25	1.64	0.15	0.05
	**Post-treatment**	21.62 ± 2.27	17.89 ± 1.49	3.73	2.48	4.97	0.001	0.49
	**Follow-up**	23.71 ± 2	18.89 ± 1.41	4.82	3.70	5.94	0.001	0.67

**SD**, standard deviation; **MD**, mean difference; ***p*-value**, level of significance; **VAS**, Visual Analog Scale; **DASH**, Disabilities of the Arm, Shoulder, and Hand scale; **SAI**, State Anxiety Inventory; **MFI**, Multidimensional Fatigue Inventory; ADLs, activity of daily living; **PG**, Pablo group**; CG**, control group. * Significant at *p* ≤ 0.017 (adjusted *p*-value).

**Table 3:** Comparison of pain, function, anxiety, fatigue, ADLs, ROM, and grip strength between pre- and post-treatment and at follow-up for PG.

Outcome	Pre-Treatment vs. Post-Treatment				Pre-Treatment vs. Follow-Up				Post-Treatment vs. Follow-Up			
	MD	95% CI		*p* Value *	MD	95% CI		*p* Value *	MD	95% CI		*p* Value *
		Lower Bound	Upper Bound			Lower Bound	Upper Bound			Lower Bound	Upper Bound	
**Pain (VAS)**	2.62	2.12	3.12	0.001	4.24	3.64	4.84	0.001	1.62	1.19	2.05	0.001
**Function (DASH)**	21.19	18.68	23.70	0.001	30.24	27.53	32.95	0.001	9.05	7.52	10.58	0.001
**Anxiety (SAI)**	6.57	4.67	8.48	0.001	11.24	9.26	13.21	0.001	4.67	3.24	6.10	0.001
**Fatigue (MFI)**	11.57	8.81	14.34	0.001	22.33	18.16	26.51	0.001	10.76	7.77	13.75	0.001
**Barthel Index for (ADLs)**	−18.33	−21.92	−14.74	0.001	−24.76	−28.13	−21.40	0.001	−6.43	−8.36	−4.50	0.001
**Flexion ROM**	−6.76	−8.22	−5.31	0.001	−9.76	−11.41	−8.12	0.001	−3	−4.21	−1.79	0.001
**Extension ROM**	−6.95	−8.73	−5.18	0.001	−8.86	−10.44	−7.28	0.001	−1.91	−2.73	−1.08	0.001
**Grip strength**	−7.72	−8.98	−6.45	0.001	−9.81	−11.02	−8.60	0.001	−2.9	−2.72	−1.47	0.001

**SD**, standard deviation; **MD**, mean difference; ***p*-value**, level of significance; **VAS**, Visual Analog Scale; **DASH**, Disabilities of the Arm, Shoulder, and Hand scale; **SAI**, State Anxiety Inventory; **MFI**, Multidimensional Fatigue Inventory; ADLs, activity of daily living; **PG**, Pablo group; **CG**, control group. * Significant at *p* ≤ 0.017 (adjusted *p*-value).

**Table 4:** Comparison of pain, function, anexity, fatigue, ADLs, ROM, and grip strength values between pre- and post-treatment and at follow-up for GG group.

Outcome	Pre-Treatment vs. Post-Treatment				Pre-Treatment vs. Follow-Up				Post-Treatment vs. Follow-Up			
	MD	95% CI		*p* Value *	MD	95% CI		*p* Value *	MD	95% CI		*p* Value *
		Lower Bound	Upper Bound			Lower Bound	Upper Bound			Lower Bound	Upper Bound	
**Pain (VAS)**	1.53	1.00	2.05	0.001	2.39	1.74	3.00	0.001	0.86	0.39	1.29	0.001
**Function (DASH)**	8.26	5.62	10.90	0.001	11.16	8.31	14.01	0.001	2.9	1.29	4.50	0.001
**Anxiety (SAI)**	1.42	−0.58	3.42	0.25	4.37	2.29	6.45	0.001	2.95	1.45	4.45	0.001
**Fatigue (MFI)**	5.84	2.93	8.75	0.001	10.73	6.35	15.13	0.001	4.89	1.75	8.04	0.001
**Barthel Index for (ADLs)**	−9.74	−13.51	−5.96	0.001	−12.11	−15.64	−8.57	0.001	−2.37	−4.40	−0.34	0.001
**Flexion ROM**	−5.68	−7.22	−4.15	0.001	−4.95	−6.68	−3.22	0.001	0.73	−0.53	2.01	0.46
**Extension ROM**	−5.26	−7.13	−3.40	0.001	−5.63	−7.29	−3.97	0.001	−0.37	−1.23	0.50	0.87
**Grip strength**	−4.68	−6.02	−3.35	0.001	−5.68	−6.96	−4.41	0.001	−1	−1.66	−0.34	0.001

**MD**, mean difference; **CI, confidence interval; *p*-value**, probability value; **VAS**, Visual Analog Scale; **DASH**, Disabilities of the Arm, Shoulder, and Hand scale; **SAI**, State Anxiety Inventory; **MFI**, Multidimensional Fatigue Inventory; ADLs, activity of daily living; **ROM,** range of motion; * Significant at *p* ≤ 0.017 (adjusted *p*-value).

## Data Availability

The raw data supporting the conclusions of this article will be made available by the authors upon request. Trial Registration: https://www.clinicaltrials.gov/ website with registration number NCT06058936.

## References

[1] Buche H., Michel A., Piccoli C., Blanc N. (2021). Contemplating or Acting? Which Immersive Modes Should Be Favored in Virtual Reality During Physiotherapy for Breast Cancer Rehabilitation. Front. Psychol..

[2] Tran K.B., Lang J.J., Compton K., Xu R., Acheson A.R., Henrikson H.J., Kocarnik J.M., Penberthy L., Aali A., Abbas Q. (2022). The global burden of cancer attributable to risk factors, 2010–2019: A systematic analysis for the Global Burden of Disease Study 2019. Lancet.

[3] Iddrisu M., Aziato L., Dedey F. (2020). Psychological and physical effects of breast cancer diagnosis and treatment on young Ghanaian women: A qualitative study. BMC Psychiatry.

[4] Chan H.K., Ismail S. (2014). Side effects of chemotherapy among cancer patients in a Malaysian general hospital: Experiences, perceptions and informational needs from clinical pharmacists. Asian Pacific J. Cancer Prev..

[5] Wirtz P., Baumann F.T. (2018). Physical Activity, Exercise and Breast Cancer—What Is the Evidence for Rehabilitation, Aftercare, and Survival? A Review. Breast Care.

[6] Fors E.A., Bertheussen G.F., Thune I., Juvet L.K., Elvsaas I.Ø., Oldervoll L., Anker G., Falkmer U., Lundgren S., Leivseth G. (2011). Psychosocial interventions as part of breast cancer rehabilitation programs? Results from a systematic review. Psychooncology.

[7] Hussein H.M., Maher A., Gabr M., Fadulelmulla I.A., Aldemery A.A., Marzouk M., Ragab M. (2024). Systematic review/Meta-analysis Impact of low-level laser therapy on upper limb lymphoedema secondary to breast cancer: A systematic review and meta-analysis. Arch. Med. Sci..

[8] Ibrahim A.A., Gabr Ali A.M.M., Fadulelmulla I.A., Ragab M.M.M., Aldemery A.A., Mohamed A.R., Dewir I.M., Hakami H.A., Hussein H.M. (2024). Using Inspiratory Muscle Training to Improve Respiratory Strength, Functional Capacity, Fatigue, and Stress in Breast Cancer Patients Undergoing Surgery. J. Multidiscip. Healthc..

[9] Cipresso P., Giglioli I.A.C., Raya M.A., Riva G. (2018). The past, present, and future of virtual and augmented reality research: A network and cluster analysis of the literature. Front. Psychol..

[10] Karamians R., Proffitt R., Kline D., Gauthier L.V. (2020). Effectiveness of virtual reality-and gaming-based interventions for upper extremity rehabilitation poststroke: A meta-analysis. Arch. Phys. Med. Rehabil..

[11] Ayed I., Ghazel A., Jaume-i-Capo A., Moya-Alcover G., Varona J., Martínez-Bueso P. (2019). Vision-based serious games and virtual reality systems for motor rehabilitation: A review geared toward a research methodology. Int. J. Med. Inform..

[12] Li L., Yu F., Shi D., Shi J., Tian Z., Yang J., Wang X., Jiang Q. (2017). Application of virtual reality technology in clinical medicine. Am. J. Transl. Res..

[13] Mouatt B., Smith A.E., Mellow M.L., Parfitt G., Smith R.T., Stanton T.R. (2020). The Use of Virtual Reality to Influence Motivation, Affect, Enjoyment, and Engagement During Exercise: A Scoping Review. Front. Virtual Real..

[14] Zhang H., Xu H., Zhang Z.X., Zhang Q. (2022). Efficacy of virtual reality-based interventions for patients with breast cancer symptom and rehabilitation management: A systematic review and meta-analysis. BMJ Open.

[15] Czech O., Siewierska K., Krzywińska A., Skórniak J., Maciejczyk A., Matkowski R., Szczepańska-Gieracha J., Malicka I. (2023). Virtual Therapy Complementary Prehabilitation of Women Diagnosed with Breast Cancer—A Pilot Study. Int. J. Environ. Res. Public Health.

[16] Chirico A., Maiorano P., Indovina P., Milanese C., Giordano G.G., Alivernini F., Iodice G., Gallo L., De Pietro G., Lucidi F. (2020). Virtual reality and music therapy as distraction interventions to alleviate anxiety and improve mood states in breast cancer patients during chemotherapy. J. Cell. Physiol..

[17] Feyzioğlu Ö., Dinçer S., Akan A., Algun Z.C., Jin A.A., Chen X.X., Zhang X.X., Chen J. (2018). Design and clinical application of rehabilitation VR system for breast cancer patients. Chin. Gen. Pract..

[18] Hartwig M. (2011). Fun and evidence-computer based arm rehabilitation with the Pablo Plus System. Prod. Profile.

[19] Chaudhary P., Hamdani N., Sharma P. (2019). The effects of visuomotor training using Pablo System on hand grip strength and wrist movements in adults and elderly. Iran. Rehabil. J..

[20] Nica A.S., Brailescu C.M., Scarlet R.G. (2013). Virtual reality as a method for evaluation and therapy after traumatic hand surgery. Annu. Rev. Cybertherapy Telemed..

[21] House G., Burdea G., Grampurohit N., Polistico K., Roll D., Damiani F., Hundal J., Demesmin D. (2016). A feasibility study to determine the benefits of upper extremity virtual rehabilitation therapy for coping with chronic pain post-cancer surgery. Br. J. Pain.

[22] Chan K.S., Zeng D., Leung J.H.T., Ooi B.S.Y., Kong K.T., Yeo Y.H., Goo J.T.T., Chia C.L.K. (2020). Measuring upper limb function and patient reported outcomes after major breast cancer surgery: A pilot study in an Asian cohort. BMC Surg..

[23] Mohammad E.B., Ahmad M., Bani Mohammad E., Ahmad M. (2019). Virtual reality as a distraction technique for pain and anxiety among patients with breast cancer: A randomized control trial. Palliat. Support. Care.

[24] Tian Q., Xu M., Yu L., Yang S., Zhang W. (2023). The Efficacy of Virtual Reality-Based Interventions in Breast Cancer-Related Symptom Management: A Systematic Review and Meta-analysis. Cancer Nurs..

[25] Freeman M.D., Gopman J.M., Salzberg C.A. (2018). The evolution of mastectomy surgical technique: From mutilation to medicine. Gland Surg..

[26] Kaidar-Person O., Offersen B.V., Boersma L.J., de Ruysscher D., Tramm T., Kühn T., Gentilini O., Mátrai Z., Poortmans P. (2021). A multidisciplinary view of mastectomy and breast reconstruction: Understanding the challenges. Breast.

[27] Hess L.M., Smith D., Cui Z.L., Montejano L., Liepa A.M., Schelman W., Bowman L. (2021). The relationship between Eastern Cooperative Oncology Group performance status and healthcare resource utilization among patients with advanced or metastatic colorectal, lung or gastric cancer. J. Drug Assess..

[28] Shao Y., Qi K., Zhou Q.-H., Zhong D.-S. (2014). Intermittent pneumatic compression pump for breast cancer-related lymphedema: A systematic review and meta-analysis of randomized controlled trials. Oncol. Res. Treat..

[29] Alotaibi N.M., Aljadi S.H., Alrowayeh H.N. (2016). Reliability, validity and responsiveness of the Arabic version of the Disability of Arm, Shoulder and Hand (DASH-Arabic). Disabil. Rehabil..

[30] Harrington S., Michener L.A., Kendig T., Miale S., George S.Z. (2014). Patient-reported upper extremity outcome measures used in breast cancer survivors: A systematic review. Arch. Phys. Med. Rehabil..

[31] Smets E.M.A., Garssen B., de Bonke B., De Haes J. (1995). The Multidimensional Fatigue Inventory (MFI) psychometric qualities of an instrument to assess fatigue. J. Psychosom. Res..

[32] Shahid A., Wilkinson K., Marcu S., Shapiro C.M. (2012). STOP, THAT and One Hundred Other Sleep Scales.

[33] Delgado D.A., Lambert B.S., Boutris N., McCulloch P.C., Robbins A.B., Moreno M.R., Harris J.D. (2018). Validation of digital visual analog scale pain scoring with a traditional paper-based visual analog scale in adults. JAAOS Glob. Res. Rev..

[34] Jensen M.P. (2003). The validity and reliability of pain measures in adults with cancer. J. Pain.

[35] dos Santos Barros V., Bassi-Dibai D., Guedes C.L.R., Morais D.N., Coutinho S.M., de Oliveira Simões G., Mendes L.P., da Cunha Leal P., Dibai-Filho A.V. (2022). Barthel Index is a valid and reliable tool to measure the functional independence of cancer patients in palliative care. BMC Palliat. Care.

[36] Morishima T., Sato A., Nakata K., Matsumoto Y., Koeda N., Shimada H., Maruhama T., Matsuki D., Miyashiro I. (2021). Barthel Index-based functional status as a prognostic factor in young and middle-aged adults with newly diagnosed gastric, colorectal and lung cancer: A multicentre retrospective cohort study. BMJ Open.

[37] Julian L.J. (2011). Measures of anxiety: State-trait anxiety inventory (STAI), Beck anxiety inventory (BAI), and Hospital anxiety and Depression scale-anxiety (HADS-A). Arthritis Care Res..

[38] Beikmoradi A., Najafi F., Roshanaei G., Esmaeil Z.P., Khatibian M., Ahmadi A. (2015). Acupressure and anxiety in cancer patients. Iran. Red Crescent Med. J..

[39] Stein J., Bishop L., Gillen G., Helbok R. (2011). Robot-assisted exercise for hand weakness after stroke: A pilot study. Am. J. Phys. Med. Rehabil..

[40] Burdea G.C., Coiffet P. (2003). Virtual Reality Technology.

[41] Feyzioğlu Ö., Dinçer S., Akan A., Algun Z.C. (2020). Is Xbox 360 Kinect-based virtual reality training as effective as standard physiotherapy in patients undergoing breast cancer surgery?. Support. Care Cancer.

[42] Atef D., Elkeblawy M.M., El-Sebaie A., Abouelnaga W.A.I. (2020). A quasi-randomized clinical trial: Virtual reality versus proprioceptive neuromuscular facilitation for postmastectomy lymphedema. J. Egypt. Natl. Cancer Inst..

[43] Chirico A., Lucidi F., De Laurentiis M., Milanese C., Napoli A., Giordano A. (2016). Virtual reality in health system: Beyond entertainment. A mini-review on the efficacy of VR during cancer treatment. J. Cell. Physiol..

